# Anogenital Distance in Turkish Newborns

**DOI:** 10.4274/jcrpe.523

**Published:** 2012-03-08

**Authors:** Alev Oğuz Kutlu

**Affiliations:** 1 Zubeyde Hanim Maternal Hospital, Department of Pediatric Endocrinology, Ankara, Turkey; kutlu64@yahoo.com.tr

**Keywords:** Anogenital distance, newborn, Turkish

## INTRODUCTION

We have measured anogenital distance (AGD) in 300 newborns delivered in Zubeyde Hanim Maternity Hospital in 2007. As in the study by Ozkan et al (1), we also used a sliding caliper for the measurements; however, in the manuscript by Ozkan et al (1), it was mentioned as tape, which needs correction.

The results of both Dr Ozkan’s study and ours seem similar. We could not give the results of measurements of anus-anterior penis base distance in male newborns and anus-clitoris base (AC) distance in female newborns. The AGD1 distance in male newborns in our study and in Ozkan et al’s study were 41.8±4.9 mm and 56±1 mm, respectively. The AC distance in female newborns in our study and in Ozkan et al’s study were 35.04±3.34 mm and 30.2±0.2 mm, respectively (unpublished data). Positive correlation of AGD1 and AC with length, head circumference and weight was also found in our study. However, no correlation was found for both the distance from the posterior base of the scrotum to the anus (ASD) in male newborns and the distance from the anus to the fourchette (AF) with same parameters. Our data suggests that the measurement of AGD1 is more reliable than ASD as the posterior border of the scrotum is an anthropometry. Romano-Riquer et al (2) also proposed that the measurement of AGD1 is more reliable than other measurement methods of AGD.

The percentile values were calculated for AGD of male and female newborns in our study. 

For female newborns, the 3^rd^, 5^th^, 10^th^, 25^th^, 50^th^, 75^th^, 90^th^, 95^th^ and 97^th^ percentile values for AF distance were found as 8.98 mm – 9.14 mm – 9.49 mm – 11.40 mm – 13.20 mm – 15.06 mm – 16.30 mm and 20.93 mm, respectively.

Percentile values for AC distance were 29.12 mm – 29.82 mm -30.94 mm – 33.00 mm – 34.50 mm - 37.20 mm - 39.39 mm and 42.19 mm, respectively.

For male newborns, the 3^rd^, 5th, 10^th^, 25^th^, 50^th^, 75^th^, 90^th^, 95^th^ and 97^th ^percentile values for ASD distance were found as 18.03 mm - 18.67 mm – 20.18 mm - 22.57 mm - 25.16 mm – 29.20 mm -32.28 mm and 36.81 mm, respectively. 

Percentile values for AGD1 distance were 32.89 mm - 33.80 mm - 35.46 mm - 38.85 mm - 40.95 mm - 45.02 mm - 48.20 mm and 51.52 mm, respectively.

I measured the stretched penile length (SPL) and found 3.77±0.35 cm as the mean value in a previous study on 514 newborns (3) and same parameter in the study by Ozkan et al (1) was reported to be 3.2±0.2 cm. Ozkan et al (1) used caliper for the measurement of SPL, whereas I used spatula. It can be considered that the difference in values could be the result of different measurement techniques. In our study, the SPL was found a bit longer than in some studies, but similar result was found in a study by Lian et al (4). In this study, SPL of Asian newborns was measured to be 3.6±0.4 cm (4).
